# Sensitivity and Cross-Reactivity Analysis of Serotype-Specific Anti-NS1 Serological Assays for Dengue Virus Using Optical Modulation Biosensing

**DOI:** 10.3390/bios15070453

**Published:** 2025-07-14

**Authors:** Sophie Terenteva, Linoy Golani-Zaidie, Shira Avivi, Yaniv Lustig, Victoria Indenbaum, Ravit Koren, Tran Mai Hoa, Tong Thi Kim Tuyen, Ma Thi Huyen, Nguyen Minh Hoan, Le Thi Hoi, Nguyen Vu Trung, Eli Schwartz, Amos Danielli

**Affiliations:** 1 Faculty of Engineering, The Institute of Nanotechnology and Advanced Materials, Bar-Ilan University, Max and Anna Webb Street, Ramat Gan 5290002, Israel; sophie.terenteva@biu.ac.il (S.T.); linoy.golani@biu.ac.il (L.G.-Z.); shiravivi@gmail.com (S.A.); 2 Central Virology Laboratory, Israel Ministry of Health, Chaim Sheba Medical Centre, Tel-HaShomer, Ramat Gan 5262000, Israel; yaniv.lustig@sheba.health.gov.il (Y.L.); viki.indenbaum@sheba.health.gov.il (V.I.); ravit.koren@sheba.health.gov.il (R.K.); 3 Department of Clinical Microbiology and Parasitology, Faculty of Medical Technology, Hanoi Medical University, Hanoi 115600, Vietnam; maihoatran@hmu.edu.vn (T.M.H.); tongkimtuyen@hmu.edu.vn (T.T.K.T.); mathihuyen@hmu.edu.vn (M.T.H.); nguyenminhoan@hmu.edu.vn (N.M.H.); lethihoi@hmu.edu.vn (L.T.H.); 4 Pasteur Institute of Ho Chi Minh City, 167 Pasteur Street, District 3, Ho Chi Minh City 722700, Vietnam; trungnv@pasteurhcm.edu.vn; 5 The Center for Geographic Medicine, Chaim Sheba Medical Centre, Tel HaShomer, Ramat Gan 5262000, Israel; eli.schwartz@sheba.health.gov.il

**Keywords:** dengue virus, zika virus, Japanese encephalitis virus, West Nile virus, non-structural protein 1, serology, cross-reactivity, optical detection

## Abstract

Dengue virus (DENV) poses a major global health concern, with over 6.5 million cases and 7300 deaths reported in 2023. Accurate serological assays are essential for tracking infection history, evaluating disease severity, and guiding vaccination strategies. However, existing assays are limited in their specificity, sensitivity, and cross-reactivity. Using optical modulation biosensing (OMB) technology and non-structural protein 1 (NS1) antigens from DENV-1–3, we developed highly sensitive and quantitative serotype-specific anti-DENV NS1 IgG serological assays. The OMB-based assays offered a wide dynamic range (~4-log), low detection limits (~400 ng/L), fast turnaround (1.5 h), and a simplified workflow. Using samples from endemic (Vietnam) and non-endemic (Israel) regions, we assessed intra-DENV and inter-*Flavivirus* cross-reactivity. Each assay detected DENV infection with a 100% sensitivity for the corresponding serotype and 64% to 90% for other serotypes. Cross-reactivity with Zika, Japanese encephalitis, and West Nile viruses ranged from 21% to 65%, reflecting NS1 antigen conservation. Our study provides valuable insights into the cross-reactivity of DENV NS1 antigens widely used in research and highlights the potential of OMB-based assays for quantitative and epidemiological studies. Ongoing efforts should aim to minimize cross-reactivity while maintaining sensitivity and explore integration with complementary platforms for improved diagnostic precision.

## 1. Introduction

Over the past 20 years, dengue virus (DENV)—a member of the *Flavivirus* genus in the *Flaviviridae* family—has spread rapidly throughout tropical and subtropical regions, becoming a significant global health concern [1,2,3]. Factors such as climate change, urbanization, and increased international travel have facilitated the spread of the virus to new areas, including Europe [4]. In 2023, over 6.5 million dengue cases and more than 7300 dengue-related deaths were reported globally [1]. Four serotypes of the virus (DENV-1–4) are known to cause dengue fever [5,6]. Following recovery, individuals develop lifelong immunity to the specific serotype they were infected with (homotypic immunity). However, cross-immunity to other serotypes is only partial and temporary (i.e., heterotypic immunity), leaving individuals susceptible to reinfection by different serotypes [7]. Such secondary infections are more likely to result in severe forms of the disease [8], manifested as dengue hemorrhagic fever (DHF), dengue shock syndrome (DSS), and/or organ impairments [9,10].

Dengue diagnostic tests detect either viral components in body fluids [11] or antiviral antibodies, specifically immunoglobulin M (IgM) and immunoglobulin G (IgG) [12]. Viral components can be directly detected by methods such as virus isolation [13], reverse transcription quantitative PCR (RT-qPCR) [5,14,15], immunohistochemistry [13], non-structural glycoprotein-1 (NS1) enzyme-linked immunosorbent assay (ELISA) [16,17], and rapid NS1-based antigen tests [17,18,19]. These methods are considered to be specific and indicate acute-phase infections [20,21]. However, *Flaviviruses* usually disappear from the blood a few days after symptoms begin, and consequently, these direct detection tests are applicable only for diagnosis of acute infection [22,23]. To confirm a previous infection, only indirect serological tests can be used since these methods are not limited to the period when the virus remains in the bloodstream. Examples are IgG ELISA tests [12,13] or plaque-reduction neutralization tests that detect virus-specific neutralizing antibodies [24]. Due to the increased risk for severe dengue in seronegative individuals who were vaccinated with the tetravalent dengue vaccine (CYD-TDV; Dengvaxia^®^, Sanofi Pasteur), the World Health Organization (WHO) recommended that pre-vaccination screening be used to ensure that only individuals with evidence of prior dengue infection are vaccinated [25,26]. Thus, effective serological tests that can ascertain previous exposure to dengue and identify the specific serotype are important tools for understanding disease severity, assessing the risk–benefit of dengue vaccination strategies, and facilitating vaccine design and deployment strategies [19,25,27,28].

The major drawbacks of serological tests are their lack of specificity [29], reduced clinical sensitivity, high cross-reactivity [30], and lengthy protocols. Most *Flavivirus* serological assays rely on the envelope (E) protein as a capture antigen [31]. Due to the high sequence similarity among E-proteins across the *Flavivirus* genus, false-positive outcomes are common [32,33]. Consequently, a positive pre-vaccination test does not reliably identify prior dengue infection because it may be caused by cross-reactivity with another *Flavivirus* infection or *Flavivirus* vaccine. To address this challenge, the NS1 protein, which is considered less cross-reactive, has been used in serological assays as a capture antigen [31,34,35]. While serological assays targeting anti-E-protein DENV IgG have been thoroughly investigated and are available commercially [31], only a few studies have investigated serological assays targeting serotype-specific anti-DENV NS1 IgG [35,36,37,38,39]. These reported assays are primarily based on ELISA and often rely on serotype-specific NS1 antigens sourced from the Native Antigen Company (Kidlington, United Kingdom). However, ELISA is inherently semi-quantitative, and there are inconsistent data regarding the clinical sensitivity and specificity of these assays, as well as their cross-reactivity both between DENV serotypes and with other *Flaviviruses*. In addition, due to overlapping clinical presentations and non-specific antibody responses during acute infection, recent reports have raised concerns regarding potential cross-reactivity between DENV serological assays and severe acute respiratory syndrome coronavirus 2 (SARS-CoV-2) [40,41]. Importantly, none of the published serotype-specific anti-DENV NS1 IgG assays report their analytical limit of detection (LoD). In fact, LoD assessments have been performed only for pan-DENV anti-NS1 IgG ELISAs. This is a significant limitation, as analytical sensitivity poses a major challenge in developing such assays. Given that NS1 is less immunogenic than the E-protein, anti-NS1 IgG antibodies are typically present at lower concentrations in DENV-infected individuals [42]. Thus, to detect anti-DENV NS1 antibodies soon after post-symptom onset (PSO) and to quantify them over a large dynamic range, a highly sensitive and quantitative detection system is needed [34].

**Figure 1:**
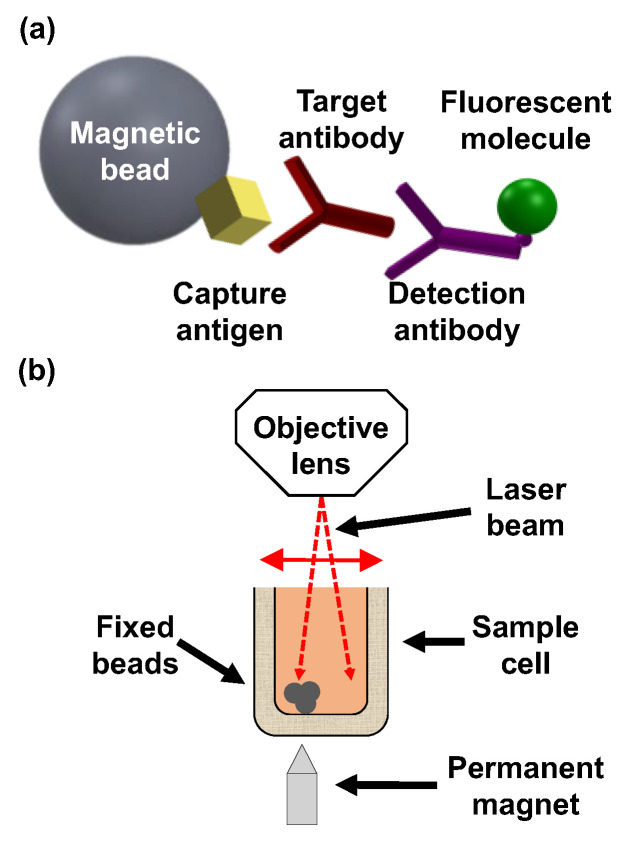
Optical modulation biosensing principles. (**a**) An OMB-based serological assay consists of a magnetic bead coated with a capture DENV NS1 antigen, target IgG antibodies, and a fluorescently labeled detection antibody. (**b**) To aggregate the magnetic beads, a small permanent magnet with a sharp tip is positioned under the sample well. To separate out the background noise of unbound fluorescent molecules, the laser beam is manipulated back and forth from the fixed beads to the background solution.

Recently, we presented a highly sensitive biosensing technology, termed optical modulation biosensing (OMB), that can detect very low concentrations of a target analyte [43,44]. In OMB, magnetic microbeads are conjugated to a specific protein that captures the target molecule. Then, a second antibody, which is fluorescently labeled, is added to form a “sandwich” assay [43] with the analyte and the capture protein (Figure 1a). To increase the sensitivity of fluorescence detection, a permanent magnet with a sharp conical tip at the bottom of the sample holder concentrates the beads from the entire solution and fixes them within the swept area of the laser beam (Figure 1b). To separate the fluorescence signal of the fluorescently labeled antibodies from the background noise caused by unbound fluorescent molecules, the laser beam is directed away from the aggregated beads towards the background solution (Figure 1b). Subtracting the background noise from the signal reduces the need for washing steps in the bioassays, shortens the detection time, and enables simple operation in non-lab settings [43]. Moreover, the signal captured from the fluorescent molecules is directly proportional to the number of target molecules in the sample. The aggregation of magnetic beads also enhances the signal, allowing quantification of the target antibody in the sample [34,45].

Using the OMB technology, we developed a high throughput biosensing system, termed OMBi, which can automatically read up to 96 samples in a conventional 96-well plate within 10 min [43,44]. In addition, we showed that the OMB technology is highly correlated with its precedent technology, termed magnetic modulation biosensing (MMB), which uses the same magnetic beads-based “sandwich” assay [43,46]. Previous results using the MMB technology to detect anti-Zika virus (ZIKV) NS1 IgM and IgG antibodies in patients’ samples showed minimal cross-reactivity with West Nile virus (WNV) and DENV (0–4%), and they demonstrated much higher clinical sensitivity (88–97%) than current state-of-the-art Euroimmun ELISA (38–74%) [34].

Here, using the OMB technology and serotype-specific NS1 antigens sourced from the Native Antigen Company, we developed serotype-specific anti-DENV NS1 IgG serological assays. We evaluated the analytical performance of each assay, including the LoD, dynamic range, and coefficient of variance (CV). To assess the clinical performance, we tested multiple patient samples from both DENV-endemic (Vietnam) and non-endemic (Israel) regions. We further examined cross-reactivity among DENV serotypes, with other *Flaviviruses* (ZIKV, Japanese encephalitis virus [JEV], and WNV), and with the non-*flavivirus* pathogen (SARS-CoV-2). Importantly, the use of ZIKV-, WNV-, and DENV-positive samples from Israeli travelers reduces the likelihood of prior DENV infections and, therefore, better represents the true cross-reactivity of the anti-DENV NS1 IgG serological assays with these *Flaviviruses*.

## 2. Materials and Methods

### 2.1. Ethical Statement

All experiments involving the collection and testing of biological materials from human subjects were performed according to guidelines and protocols approved by the institutional review boards of Hanoi Medical University (IRB-VN01.001/IRB00003121/FWA 00004148) and Sheba Medical Center in Israel (IRB 4420-17).

### 2.2. Sample Collection

Serum samples were collected from 14 DENV-1-positive and 60 DENV-2-positive patients. Of those 74 patients, 8 DENV-1 and 51 DENV-2 samples were obtained from Hanoi Medical University in Vietnam. The remaining six DENV-1 and nine DENV-2 samples were collected from Israeli travelers returning from DENV-endemic countries. All samples were initially confirmed positive for DENV-1 or DENV-2 by qRT-PCR at the time of hospitalization. The samples analyzed in this study were obtained from follow-up blood draws. Specifically, all 14 DENV-1 samples were collected from day seven PSO onward (Table S1), and all 60 DENV-2 samples were collected from day eight PSO onward (Table S2). Samples from Israeli travelers were further confirmed positive using antigen tests (rapid NS1 or ELISA) and/or ELISA-based IgM/IgG tests (Panbio™ Dengue IgM Capture ELISA and Panbio™ Dengue IgG Indirect ELISA, Catalog No. 01PE20 and 01PE30, Abbott Diagnostics Korea Inc., Seoul, Republic of Korea).

Additionally, Hanoi Medical University in Vietnam provided 28 serum samples from patients who tested positive for JEV, confirmed by ELISA IgM assays (Table S3). The National Center for Zoonotic Viruses at the Central Virology Laboratory of the Ministry of Health, Sheba Medical Center (Israel), provided 23 WNV-positive serum samples, 26 ZIKV-positive serum samples, 22 SARS-CoV-2-positive serum samples, and 35 serum samples from healthy individuals. The WNV-positive patients were confirmed positive using either RT-qPCR or ELISA IgM and IgG assays (Table S4). The Zika-positive samples were confirmed positive by either RT-qPCR or neutralization tests (Table S5). The SARS-CoV-2-positive serum samples were collected at least 14 days after a positive RT-qPCR test and were further confirmed as IgG-positive by ELISA [45] (Table S6).

Patient information—including age, gender, country of origin, country of acquisition, and the day the sample was taken after the onset of symptoms—was obtained from the electronic medical record. All the samples (50 μL each) were stored at −80 °C, delivered to Bar-Ilan University on dry ice, and then thawed once for the OMB-based assay.

### 2.3. Analytical Performance of the OMB-Based Anti-DENV-1–2 NS1 IgG Assays

The OMB-based anti-DENV-1–3 NS1 IgG serological assays were constructed using a procedure that has been described previously [34]. Briefly, each assay consisted of approximately 25,000 tosylactivated magnetic beads (M-280; Thermo Fisher, Carlsbad, CA, USA) that were pre-conjugated to either recombinant DENV-1-, DENV-2-, or DENV-3-NS1 antigen protein (DENV1-NS1, DENV2-NS1, DENV3-NS1; The Native Antigen Company, Kidlington, United Kingdom).

To characterize the analytical performance (i.e., the analytical sensitivity, LoD, dynamic range, and CV) of the OMB-based anti-DENV-1–2 NS1 IgG serological assays, we conducted a full dose–response experiment. The beads of each assay were incubated for 60 min at room temperature with mouse anti-DENV-1/2 NS1 IgG antibodies (MAB12132, MAB12133; The Native Antigen Company, Kidlington, UK) at one of a range of concentrations: 0, 1 × 10^1^, 1 × 10^2^, 1 × 10^3^, 1 × 10^4^, 1 × 10^5^, 1 × 10^6^, and 1 × 10^7^ ng/L. After the initial incubation, the beads were washed, followed by incubation with a fluorescently labeled detection antibody, AlexaFluor™-532 goat anti-mouse IgG H&L (A-11002, Thermo Fisher, Carlsbad, CA, USA). To remove unbound antibodies, a single buffer replacement was performed after the final incubation. All incubation steps were executed at room temperature within a 96-well plate positioned on a rotary shaker. Each sample was divided into two equal portions of 100 μL. For each concentration of the target mouse anti-DENV NS1 IgG antibody, triplicate samples were loaded into three wells of a 96-well plate and measured using the OMBi system (*n* = 3). For the blank concentration, six samples were prepared and measured (*n* = 6).

The reaction and washing buffer solution consisted of phosphate-buffered saline (×1) (PBS), 10 mg/mL bovine serum albumin (BSA; VWR), and 0.05% Tween 20 (Sigma-Aldrich Corporation, St. Louis, MO, USA).

### 2.4. Clinical Sensitivity and Specificity of the OMB-Based Anti-DENV-1–3 NS1 IgG Serological Assays

**Figure 2:**
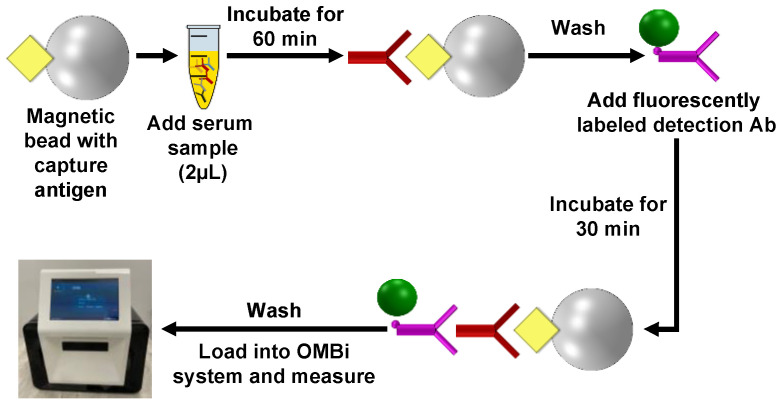
Clinical evaluation of the OMB-based anti-DENV-1–3 NS1 IgG serological assays. Magnetic beads with a capture antigen (e.g., DENV1-NS1) are mixed with 2 µL of the clinical serum sample and incubated for 60 min. Following a single washing step, fluorescently labeled detection antibody is added and incubated for 30 min. After an additional wash, the plate is loaded into the OMBi system for measurement.

To evaluate the clinical sensitivity and specificity of the OMB-based anti-DENV-1–3 NS1 IgG serological assays, we used a protocol described previously [34]. For each serotype-specific assay (e.g., anti-DENV-1 NS1 IgG assay), the conjugated beads were placed in a 96-well plate, mixed with 2 µL of the clinical serum sample, and incubated for 60 min at room temperature. Following a single wash, a fluorescently labeled detection antibody (donkey F (ab′)2 anti-human IgG H&L (phycoerythrin [PE]), ab7005; Abcam plc., Cambridge, UK) was added and incubated for 30 min at room temperature, forming a “sandwich” assay. After an additional wash, the plate was loaded into the OMBi system (Software version OMB-1.0.0) for measurement (Figure 2).

To determine the cutoff for each of the anti-DENV-1–3 NS1 IgG serological assays, we tested 35 healthy serum samples. The cutoff was calculated as three standard deviations from the average signal from healthy patients. To evaluate clinical sensitivity, specificity, and cross-reactivity within serotypes of the anti-DENV-1 NS1 IgG serological assay, we tested 14 DENV-1-positive and 60 DENV-2-positive serum samples that had been collected from day seven PSO onward. For both the anti-DENV-2 and anti-DENV-3 NS1 serological assays, we tested 11 DENV-1-positive and 29 DENV-2-positive serum samples. Due to a lack of sufficient DENV-3-positive samples, the anti-DENV-3 NS1 serological assay was tested only for specificity and cross-reactivity with DENV-1 and DENV-2.

### 2.5. Cross-Reactivity of the OMB-Based Anti-DENV-1 and Anti-DENV-3 NS1 IgG Serological Assays with WNV, JEV, ZIKV, and SARS-CoV-2

To determine the cross-reactivity between the DENV-1 NS1 and other *Flaviviruses*, we tested the anti-DENV-1 NS1 IgG serological assay using 23 WNV-positive, 28 JEV-positive, and 26 ZIKV-positive samples. To evaluate the differences in cross-reactivity of the anti-ZIKV NS1 IgG antibodies with different DENV NS1 antigens, we further tested the same 26 ZIKV-positive samples using the anti-DENV-3 NS1 IgG serological assay. Additionally, to evaluate potential cross-reactivity with SARS-CoV-2, we tested 22 serum samples from patients with confirmed SARS-CoV-2 infection using the anti-DENV-1 NS1 IgG serological assay.

### 2.6. Data Analysis

The clinical sensitivities of the anti-DENV-1 and anti-DENV-2 NS1 IgG serological assays were calculated as the percentage of DENV-1- or DENV-2-positive patients correctly identified as positive by the assay. Specificity was calculated as the percentage of healthy individuals correctly identified as negative by the assay [47,48,49]. Cross-reactivity was assessed as the percentage of samples from other DENV serotypes or other non-DENV *Flaviviruses* that yielded positive results, indicating unintended detection of non-target antibodies [47,48]. Statistical analyses were performed using GraphPad Prism 10 (version 10.5.0.774, GraphPad Software, Inc., San Diego, CA, USA). The LoD was calculated using previously described parametric tests [43,50]. Briefly, the limit of the blank (LoB) was calculated from the blank measurements for each target as follows:

(1) $$LoB={µ}_{blank}+1.645\times \sigma_{blank},$$

where $\mu_{blank}$ is the mean, and $\sigma_{blank}$ is the standard deviation. The LoD was calculated from a pool of all low-concentration measurements and standard deviations

(2) $$LoD=LoB+1.645\times \sigma_{pooled},$$

where $\sigma_{pooled}$ is the pooled standard deviation of low-concentration measurements [50].

## 3. Results

### 3.1. Analytical Performance of the OMB-Based Anti-DENV-1–2 NS1 IgG Serological Assays

**Figure 3:**
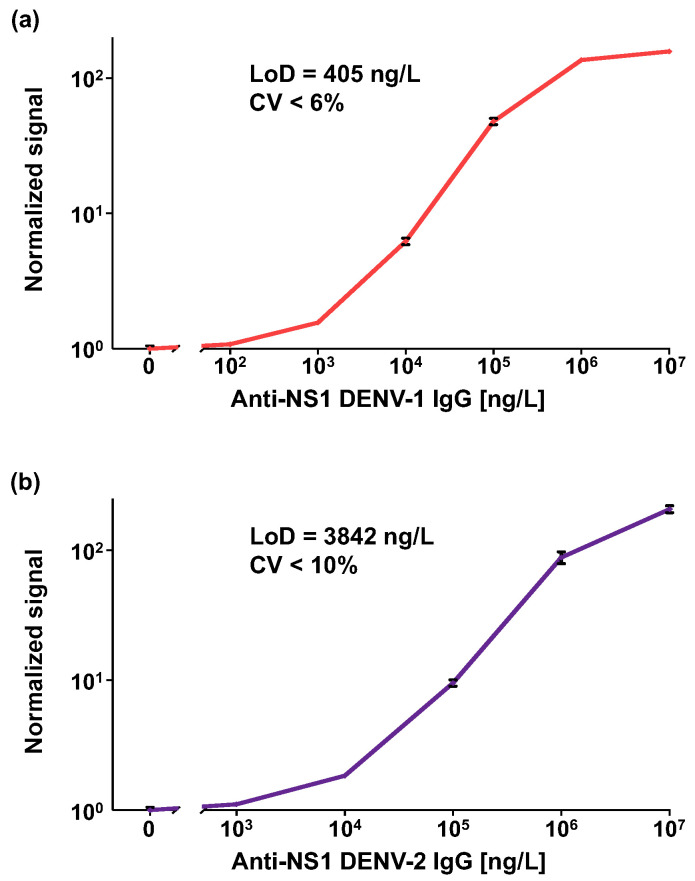
Analytical performance of OMB-based anti-DENV-1–2 NS1 IgG serological assays. (**a**) Dose–response of a recombinant anti-DENV-1 NS1 IgG antibody using the OMB-based anti-DENV-1 NS1 IgG serological assay, and (**b**) dose–response of a recombinant anti-DENV-1 NS1 IgG antibody using the OMB-based anti-DENV-2 NS1 IgG serological assay. The limits of detection (LoDs) are 405 ng/L and 3842 ng/L, and the coefficients of variance (CV) are 6% and 10%, respectively. The error bars represent the standard error of three (n=3) experiments performed on different days.

Figure 3 shows the dose–response curves for the anti-DENV-1 and anti-DENV-2 NS1 IgG assays. The calculated LoDs are 405 ng/L and 3842 ng/L, with coefficients of variation of less than 6% and 10%, respectively. For both assays, the dynamic range spans approximately 4-log.

### 3.2. Evaluation of Clinical Sensitivity and Specificity of the OMB-Based Anti-DENV-1–3 NS1 IgG Serological Assays

**Figure 4:**
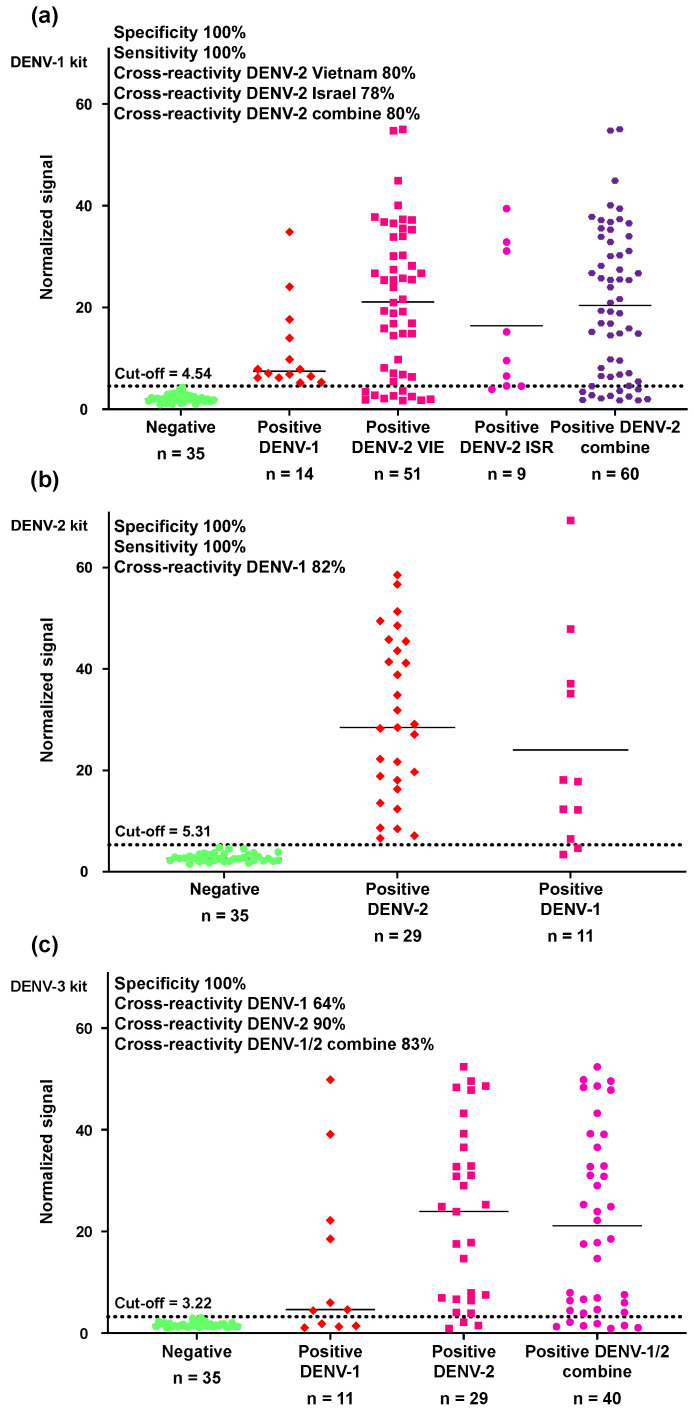
Clinical sensitivity, specificity, and cross-reactivity within serotypes of the OMB-based anti-DENV-1–3 NS1 IgG serological assays. Clinical sensitivity, specificity, and cross-reactivity within serotypes of the OMB-based (**a**) anti-DENV-1, (**b**) anti-DENV-2, and (**c**) anti-DENV-3 NS1 IgG serological assays (marked as DENV-1 kit, DENV-2 kit, and DENV-3 kit, respectively). The cutoff for each assay was determined as three standard deviations over the average signal from the healthy patients.

The anti-DENV-1 NS1 IgG serological assay demonstrated 100% clinical sensitivity for DENV-1 and 100% specificity relative to healthy controls (Figure 4a, Table S1). Based on samples collected in Vietnam (an endemic country) and Israel, the cross-reactivities of the anti-DENV-1 NS1 IgG serological assay with DENV-2 were 80% and 78%, respectively. For combined samples from Vietnam and Israel, the total cross-reactivity was 80% (Figure 4a, Tables S1 and S2). Similarly, the anti-DENV-2 NS1 IgG serological assay had 100% clinical sensitivity for DENV-2 and 100% specificity relative to healthy controls (Figure 4b, Tables S1 and S2), but the cross-reactivity of the anti-DENV-2 NS1 IgG serological assay with DENV-1 was 82%.

Due to a lack of DENV-3-positive samples, we could not determine the clinical sensitivity of the anti-DENV-3 NS1 IgG serological assay. However, the clinical specificity relative to healthy controls was 100% (Figure 4c, Tables S1 and S2), and the cross-reactivities with DENV-1 and DENV-2 were 64% and 90%, respectively. The combined cross-reactivity with DENV-1 and DENV-2 was 83%. The results of the OMB-based anti-DENV NS1 IgG serological assays in different serum panels, along with their calculated sensitivities, specificities, and cross-reactivities, are summarized in Table 1. For each serum panel (i.e., each column), the numbers in each row represent the number of samples that were identified as IgG-positive by the anti-DENV-1–3 NS1 IgG serological assay (individually titled as DENV-1 kit, DENV-2 kit, and DENV-3 kit).

### 3.3. Cross-Reactivity of the OMB-Based Anti-DENV-1 NS1 IgG Serological Assay with WNV, JEV, ZIKV, and SARS-CoV-2

**Figure 5:**
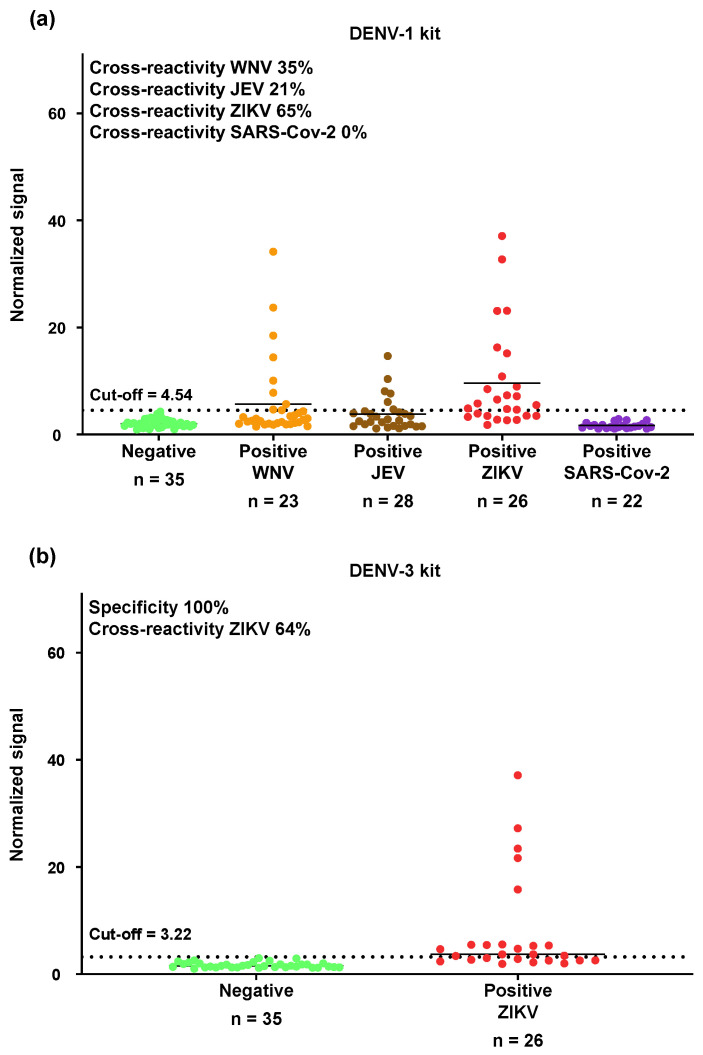
Cross-reactivity analysis of the OMB-based anti-DENV NS1 IgG serological assays with other *Flaviviruses* and SARS-CoV-2. (**a**) Cross-reactivity of the OMB-based anti-DENV-1 NS1 IgG serological assay (marked as DENV-1 kit) with WNV, JEV, ZIKV, and SARS-CoV-2. (**b**) Cross-reactivity of the OMB-based anti-DENV-3 NS1 IgG serological assay (marked as DENV-3 kit) with ZIKV. The cutoff for each assay was determined as three standard deviations over the average signal from the healthy patients.

The cross-reactivities of the OMB-based anti-DENV-1 NS1 IgG serological assay with WNV, JEV, and ZIKV were 35%, 21%, and 65% (Figure 5a, Table 1 and Tables S3–S5). Importantly, no cross-reactivity was observed when the DENV-1 assay was tested with SARS-CoV-2-positive samples (0% cross-reactivity; Table S6). To confirm the high cross-reactivity of the DENV-1 serological assay with ZIKV, we tested the same ZIKV samples using the anti-DENV-3 NS1 IgG serological assay and observed a similar cross-reactivity rate of 62% (Figure 5b, Table 1). The total number of concordant results (positive and negative) between the cross-reactivity rates of the DENV-1 and DENV-3 assays with ZIKV was 21 out of 26, resulting in an overall agreement of 81%. Among the 17 ZIKV-positive samples that were falsely identified as DENV-1-positive by the DENV-1 kit, 14 were also falsely identified as DENV-3-positive by the DENV-3 kit (Table S5).

## 4. Discussion

Serotype-specific DENV serological assays are critical for understanding an individual’s history of dengue infection and assessing the potential severity of the disease. Additionally, these assays can help identify individuals who may benefit from the dengue vaccine and those for whom vaccination may not be necessary, thereby aiding in the design and deployment of dengue vaccination strategies [36].

Most commercially available DENV IgG serological assays use the E-protein as the capture antigen. However, due to the high similarity of the E-protein across *Flaviviruses*—and its even higher similarity within DENV serotypes—these assays often exhibit high cross-reactivity and low specificity. Recently, several studies have focused on the more specific NS1 protein for anti-DENV NS1 IgG serological assays (Table 2). For example, Tsai et al. (2017) [39] developed an anti-DENV-1 NS1 IgG ELISA and demonstrated its ability, in combination with anti-ZIKV NS1 IgM and IgG ELISAs, to distinguish primary ZIKV infections from ZIKV with prior DENV exposure and secondary DENV infections [39]. Nascimento et al. (2018) [35] developed a pan-DENV anti-NS1 IgG ELISA test targeting all serotypes using the same test. The assay had an LoD of 2.33 EU/mL and a 2-log dynamic range. Based on very few clinical samples (3–4), the cross-reactivity with ZIKV and WNV was high [35]. Galula et al. (2021) [37] developed another pan-DENV anti-NS1 IgG ELISA with an LoD of ~1000–2000 ng/L and reported a clinical sensitivity of 91.7% and specificity of 82.3%. To investigate the immunoglobulin responses of patients with dengue fever and dengue hemorrhagic fever, Jayathilaka et al. (2018) [36] developed anti-DENV-1 and anti-DENV-2 NS1 IgG ELISAs and measured the kinetics of anti-NS1 IgG antibodies in acute secondary dengue infection. However, the analytical and clinical sensitivity of the assay were not reported. Tyson et al. (2019) [38] developed anti-DENV-1–4 NS1 IgG ELISAs with sensitivities of 92–100% (for samples > 3 months post-symptom onset) and cross-reactivities ranging from 23.8% to 100% within serotypes. By combining these ELISAs with an anti-ZIKV NS1 IgG ELISA, they were able to distinguish between ZIKV with previous DENV and secondary DENV infections, with sensitivities of ~91.7–94.1% and specificities of 87.0–95.0%. Matsunaga et al. (2021) [51] introduced four DNA aptamers targeting DENV NS1 proteins in a competitive ELISA format to detect serotype-specific IgG. The analytical sensitivity of the assay was not measured, but the assay could detect anti-DENV NS1 IgG antibodies in DENV-positive patients with primary infection from day 17 PSO onward. The authors noted discrepancies between their competitive ELISA and standard methods, such as RT-qPCR, likely due to past infections, and emphasized the need for further research. While these methods show promise, none of the serotype-specific anti-DENV NS1 IgG assays reported its analytical performance, and evidence of their specificity and cross-reactivity with other *Flaviviruses* remains mixed. Moreover, as these assays are based on ELISA, they are time-consuming (~4 h), require multiple washing steps, and rely on enzymatic reactions, limiting their dynamic range (~2-log) and rendering them only semi-quantitative. Accordingly, their applicability in quantitative studies is limited. Table 2 summarizes these studies.

One of the main challenges in developing anti-DENV NS1 IgG serological assays is achieving high analytical sensitivity. In contrast to previous studies that reported LoD values only for pan-DENV anti-NS1 IgG ELISA assays [35,37], we determined the LoD for our serotype-specific OMB-based assays targeting anti-DENV-1 and anti-DENV-2 NS1 IgG antibodies using the CLSI EP17-A2 guideline [50]. This approach, which defines the LoB and LoD based on replicate measurements of blank and low-concentration samples, provides improved statistical rigor, reproducibility, and diagnostic relevance. Compared to previously reported ELISA-based anti-DENV NS1 IgG tests—whether pan-serotype or serotype-specific—the OMB-based anti-DENV-1 NS1 IgG assay demonstrates a significantly broader dynamic range (~4-log vs. ~2-log) and a comparable or improved LoD (~400 ng/L vs. ~1000–2000 ng/L) [35,37]. In addition, the OMB-based assay offers a much shorter turnaround time (1.5 h vs. ~4 h) and requires fewer washing steps—just two compared to the 7–10 steps typically needed for ELISA—making it both simpler and less labor-intensive. Another key advantage of the OMB-based assay is its resistance to data drift caused by delayed optical reading. In ELISA tests, high background noise must be mitigated by terminating the enzyme–substrate reaction with a stop solution, followed by immediate optical reading. Any delay can lead to data drift and false results [52]. In contrast, the OMB-based assay maintains stable fluorescence signals even with delayed readings, providing more reliable outcomes [45]. Furthermore, while ELISA is inherently semi-quantitative, the OMB-based assay provides fully quantitative results, enhancing its utility for precise and reproducible measurements.

The OMB-based anti-DENV-1 and anti-DENV-2 NS1 serological assays demonstrated 100% clinical sensitivity for DENV-1- and DENV-2-positive samples, respectively, and 100% specificity relative to samples from healthy populations. Although the clinical sensitivity and specificity of the OMB-based anti-DENV NS1 IgG serological assays are comparable to those reported by Tyson et al. (2019) [38], the cross-reactivity within serotypes is slightly higher. For example, the cross-reactivity of the DENV-2 assay with DENV-1-positive samples, and vice versa, was 80–82% (Figure 4). Although previous studies have attributed high cross-reactivity within serotypes to previous DENV infections [38], this study observed similarly elevated cross-reactivity in samples from DENV-positive patients in both an endemic country (Vietnam) and a non-endemic country (i.e., Israeli travelers), for whom the likelihood of previous DENV infection is low. Furthermore, despite the absence of DENV-3-positive samples, the DENV-3 assay exhibited notable cross-reactivity, with rates of 64% for DENV-1-positive samples and 90% for DENV-2-positive samples. The high cross-creativity within serotypes in this study may be attributed to the use of DENV-positive samples collected on days 7–18 PSO—a timeframe during which the affinity and specificity of IgG antibodies are relatively low—resulting in broader binding and less specificity. In contrast, Tyson et al. primarily analyzed post-convalescent samples, which likely contained more mature antibodies with higher specificity [38].

**Figure 6:**
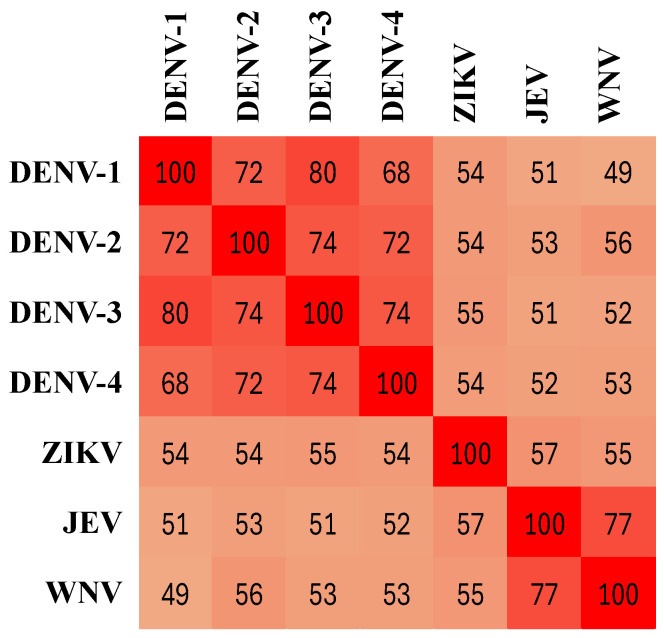
Non-structural glycoprotein-1 (NS1) sequence similarity among dengue virus serotypes (DENV-1–4), Zika virus (ZIKV), Japanese encephalitis virus (JEV), and West Nile virus (WNV). The numbers represent the percent identity as calculated by the basic local alignment search tool (BLAST, version 1.4.0, see Supplementary Materials). The color gradient corresponds to percent identity, with dark red indicating high similarity (close to 100%) and light red indicating low similarity (e.g., ~50%).

Because the NS1 protein of *Flaviviruses* is highly conserved, antibodies directed against the NS1 of a specific *Flavivirus* are likely to cross-react with the NS1 protein of other *Flaviviruses* [36,53]. The degree of conservation across different serotypes and *Flaviviruses* can be illustrated by the similarity of their NS1 amino acid sequences (Figure 6). The high percentage of conserved sequences between DENV and other *Flaviviruses*—and even higher conservation within DENV serotypes—suggests the presence of shared epitopes among these *Flaviviruses*. Here, we found that the cross-reactivities of the OMB-based anti-DENV-1 NS1 IgG serological assay with ZIKV-, JEV-, and WNV-positive patients were 65%, 21%, and 35%, respectively. It is important to note that the ZIKV- and WNV-positive samples were obtained from Israeli travelers, where the likelihood of previous DENV infection is low. In addition, the JEV-positive samples were confirmed positive by a JEV ELISA IgM test, but there is no information regarding the day the samples were collected PSO. Thus, the IgG levels could have been low, suggesting that the actual cross-reactivity between DENV-1 and JEV could be higher.

Despite the high structural similarity of NS1 proteins among *Flaviviruses*, actual cross-reactivity must be experimentally validated, preferably using positive samples from travelers returning from endemic regions, as they are less likely to have previous DENV infections. Interestingly, while the cross-reactivity of the DENV-1 NS1 antigen with anti-ZIKV NS1 IgG antibodies is high, our previous findings using the ZIKV NS1 antigen from the Native Antigen Company as a capture antigen in anti-ZIKV NS1 IgM and IgG serological assays demonstrated minimal cross-reactivities (0–4%) with WNV and DENV [34]. Similarly, Steinhagen et al. (2016) reported low cross-reactivity between ZIKV and DENV in Euroimmun’s anti-ZIKV NS1 IgG ELISA [33]. This asymmetry in cross-reactivity may be attributed to differences in the panels of ZIKV-positive and DENV-positive samples used in these studies. For instance, most of the Zika samples in our previous study were collected from day 28 PSO or later, when the affinity of anti-ZIKV NS1 IgG antibodies was high. This resulted in a significant signal difference between healthy patients’ samples and ZIKV-positive samples [34]. Consequently, the cutoff for the OMB-based anti-ZIKV NS1 IgG serological assay was set at ~12 standard deviations above the mean signal from healthy patients’ samples. While maintaining high clinical sensitivity, this cutoff effectively minimized false-positive signals due to cross-reactivity with DENV-positive samples [34]. In contrast, the DENV-positive samples used in the current study were collected 8–15 days PSO when IgG antibody affinity was lower. As a result, the cutoff was set at three standard deviations above the blank measurements, making it more challenging to prevent false-positive signals from ZIKV-positive samples.

Considering recent concerns about potential serological cross-reactivity between SARS-CoV-2 and DENV, we evaluated whether the OMB-based anti-DENV-1 NS1 IgG assay cross-reacts with antibodies generated in response to SARS-CoV-2. None of the 22 serum samples from RT-qPCR-confirmed SARS-CoV-2 patients yielded a positive result, indicating 0% cross-reactivity. This finding is consistent with Lustig et al. (2020) [40], who reported cross-reactivity in DENV serological assays targeting the E protein but not the NS1 protein. In contrast, Cheng et al. (2022) [41] showed that antibodies against the receptor-binding domain (RBD) of the SARS-CoV-2 spike protein subunit 1 (S1) can cross-react with DENV NS1. Our results provide experimental evidence for the specificity of the OMB-based NS1 assay and suggest that its design effectively minimizes non-specific interactions, including those from unrelated viral infections such as SARS-CoV-2.

It is important to note that while serotype-specific anti-DENV antibodies can serve as markers of prior exposure, their presence alone does not guarantee clinical protection. Recent studies have shown that the correlation between neutralizing antibody levels and protective immunity remains unclear and may vary across individuals and serotypes [54]. Indeed, two recently approved live-attenuated vaccines—Dengvaxia^®^ (CYD-TDV) and Qdenga^®^ (TAK-003)—were designed to elicit neutralizing antibodies against all four DENV serotypes, yet field studies have demonstrated variable and, in some cases, low efficacy against certain serotypes [55,56]. Therefore, although quantitative assays like the one presented here can provide valuable insights into exposure history and immune profiling, they may be interpreted alongside other immunological and clinical parameters when evaluating vaccine efficacy.

Limitations of our study include the relatively small sample size for DENV-1-positive cases (11–14 samples) and the absence of DENV-3- and DENV-4-positive samples. To further validate our findings, additional testing with a larger and more diverse sample set is necessary. The highly sensitive and quantitative nature of the OMB-based serological assays provides an opportunity for future studies to investigate potential differences in immune responses among individuals with varying disease severities, vaccination types, and diverse geographic or ethnic backgrounds. While the OMB-based assays demonstrated high clinical sensitivity, the observed cross-reactivity rates of ~80% between DENV serotypes indicate that the current assays are not suitable for identifying the infecting serotype in individual patients. Future research should focus on reducing cross-reactivity while maintaining the rapid, highly sensitive, and quantitative performance of the OMB platform. For example, similar to the approach described by Tsai et al. (2017) [39], the OMB-based anti-DENV-1–2 NS1 IgG serological assays could be used alongside OMB-based serological assays for other *Flaviviruses* [34]. This integrated approach could provide rapid, user-friendly assays capable of distinguishing between primary DENV infections, other *Flavivirus* infections in individuals with prior DENV exposure, and secondary DENV infections [39]. Additionally, block-of-binding techniques [57]—which benefit from the broad dynamic range and quantitative capabilities of the OMB platform—could offer a complementary strategy for improving serotype discrimination.

## 5. Conclusions

Here, using OMB technology and NS1 antigens from DENV-1–3, we demonstrated quantitative and rapid anti-DENV-1–3 NS1 IgG serological assays with high analytical and clinical sensitivity. Compared to ELISA, the OMB-based serological assays offer quantitative results, a broader dynamic range, comparable or better LoDs, shorter turnaround times, and a simpler, more stable protocol. Using clinical samples from both endemic and non-endemic regions, we evaluated the cross-reactivity of the assays among DENV serotypes and with other *Flaviviruses*, including ZIKV, JEV, and WNV. While the assays demonstrated high clinical sensitivity and specificity relative to samples from healthy individuals, significant cross-reactivity was observed, consistent with the conserved nature of the NS1 protein. Notably, no cross-reactivity was detected in samples from SARS-CoV-2-infected individuals, suggesting limited non-specific binding from unrelated viral infections. Because the DENV NS1 antigens employed in this study—sourced from the Native Antigen Company—are commonly utilized by researchers in the field [35,36,37,38,39,51], the information derived from this study regarding their cross with JEV, WNV, and ZIKV has significant value [35].

Overall, the OMB-based serological assays are promising tools for epidemiological studies, vaccine evaluation, and immunological research. Future work should focus on reducing cross-reactivity while maintaining the assays’ high sensitivity and quantitative accuracy. These advances will support global efforts in DENV control, vaccination strategies, and public health decision-making.

## Figures and Tables

**Table 1:** Results of OMB-based anti-DENV NS1 IgG serological assays in different serum panels. Abbreviations: ISR—Israel, VIE—Vietnam, DENV—dengue virus, WNV—West Nile virus, JEV—Japanese encephalitis virus, ZIKV—Zika virus, SARS-CoV-2—severe acute respiratory syndrome coronavirus 2.

DENV Kit	No. of IgG^+^/Total Samples (%) in Different Serum Panels									
	Naive	Positive DENV-1	Positive DENV-2 ISR	Positive DENV-2 VIE	Positive DENV-2 Combine	Positive DENV-1 +DENV-2 Combine	Positive WNV	Positive JEV	Positive ZIKV	Positive SARS-CoV-2
DENV-1 kit	0/35 (0)	14/14 (100)	7/9 (78)	41/51 (80)	48/60 (80)	62/74 (84)	8/23 (35)	6/28 (21)	17/26 (65)	0/22 (0)
DENV-2 kit	0/35 (0)	9/11 (82)	9/9 (100)	20/20 (100)	29/29 (100)	38/40 (95)	Not tested	Not tested	Not tested	Not tested
DENV-3 kit	0/35 (0)	7/11 (64)	8/8 (100)	18/21 (86)	26/29 (90)	33/40 (83)	Not tested	Not tested	16/26 (62)	Not tested

**Table 2:** Studies detecting anti-DENV NS1 IgG antibodies.

Paper Author	Assay and Technology Type	Limit of Detection
Tsai et al. (2017) [39]	Anti-DENV-1 NS1 IgG ELISA	Not reported
Nascimento et al. (2018) [35]	Pan-DENV anti-NS1 IgG ELISA	2.33 EU/mL
Jayathilaka et al. (2018) [36]	Anti-DENV-1 and anti-DENV-2 NS1 IgG ELISA	Not reported
Tyson et al. (2019) [38]	Anti-DENV-2–4 NS1 IgG ELISA and Pan-DENV anti-NS1 IgG ELISA	Not reported
Galula et al. (2021) [37]	Pan-DENV anti-NS1 IgG ELISA	Indirect ELISA: 1030–1260 ng/L GAC-ELISA: 1380–1870 ng/L
Matsunaga et al. (2021) [51]	Anti-DENV-1–4 NS1 IgG ELISA and Pan-DENV anti-NS1 IgG ELISA Competitive ELISA	Not reported

## Data Availability

Data will be made available on request.

## Supplementary Materials

The following supporting information can be downloaded at https://www.mdpi.com/article/10.3390/bios15070453/s1, Table S1: Characteristics of DENV-1-positive samples; Table S2: Characteristics of DENV-2-positive samples; Table S3: Characteristics of JEV-positive samples; Table S4: Characteristics of WNV-positive samples; Table S5: Characteristics of ZIKV-positive samples; List of NS1 sequences used for BLAST analysis; Table S6: Characteristics of SARS-Cov-2-positive samples* (total number of samples *n* = 22).

## Abbreviations

The following abbreviations are used in this manuscript:

OMB	Optical modulation biosensing
LoD	Limit of detection
LoB	Limit of blank
CV	Coefficient of variance
DENV	Dengue virus
WNV	West Nile virus
ZIKV	Zika virus
JEV	Japanese encephalitis virus
ELISA	Enzyme-linked immunosorbent assays
SARS-CoV-2	Severe acute respiratory syndrome coronavirus 2
RBD	Receptor-binding domain
