# Supplementary material for: Sensitivity and Cross-Reactivity Analysis of Serotype-Specific Anti-NS1 Serological Assays for Dengue Virus Using Optical Modulation Biosensing
Source: Biosensors (Basel). 2025 Jul 14;15(7):453. doi: 10.3390/bios15070453 (PMC12293919; doi:10.3390/bios15070453)
Supplement: Supplementary file 1 [file biosensors-15-00453-s001.zip › biosensors-3669462-supplementary.pdf]

Table S1: Characteristics of DENV-1-positive samples* **(total number of samples $n = 14$)**. Abbreviations: F – female, M – Male, Pos – Positive, Neg – Negative.

#	Patient #	Country of possible exposure	Gender / age(yr)	Time from onset (days)	ELISA DENV IgM	ELISA DENV IgG	OMB anti-NS1 DENV-1 IgG	OMB anti-NS1 DENV-2 IgG	OMB anti-NS1 DENV-3 IgG
1	10.3	Vietnam	M/17	10		Pos	Pos	Pos	Pos
2	27.2	Vietnam	F/27	7		Pos	Pos	Neg	Neg
3	56.3	Vietnam	F/-	8			Pos	Pos	Pos
4	67.3	Vietnam	M/50	8			Pos	Pos	Pos
5	164.3	Vietnam	M/21	11			Pos	Pos	Pos
6	203.2	Vietnam	F/16	8			Pos	Pos	Neg
7	203.3	Vietnam	F/16	11			Pos	Pos	Pos
8	211.2	Vietnam	F/28	8			Pos	Pos	Pos
9	732/18†	Philippines	M/31		Pos	Pos	Pos	Pos	Pos
10	735/18†	Philippines	F/28		Pos	Pos	Pos	Pos	Neg
11	286/16†		M/33		Pos	Pos	Pos		
12	977/16†		F/73		Pos	Pos	Pos	Neg	Neg
13	648/16†	Brazil	F/42		Pos	Neg	Pos		
14	1260/17†		M/27		Pos	Pos	Pos		

*Patients were initially confirmed positive for DENV-1 by qRT-PCR at the time of hospitalization. The samples analyzed in this study were obtained from follow-up blood draws collected more than 7 days after symptom onset.

†Israeli traveler

Table S2: Characteristics of DENV-2-positive samples* **(total number of samples $n = 60$)**. Abbreviations: F – female, M – Male, Pos – Positive, Neg – Negative.

#	Patient #	Country of possible exposure	Gender / age(yr)	Time from onset (days)	ELISA DENV IgG	OMB anti-NS1 DENV-1 IgG	OMB anti-NS1 DENV-2 IgG	OMB anti-NS1 DENV-3 IgG
1	11.3	Vietnam	M/46	9	Pos	Pos		
2	73.2	Vietnam	F/29	8	Pos	Pos	Pos	Pos
3	76.2	Vietnam	F/54	8	Pos	Pos	Pos	Pos
4	86.2	Vietnam	F/29	8		Pos		
5	89.3	Vietnam	F/46	10	Pos	Pos	Pos	Pos
6	90.3	Vietnam	M/17	10	Pos	Pos		Pos
7	91.2	Vietnam	M/72	10		Neg		
8	91.3	Vietnam	M/72	13	Pos	Pos		
9	93.1	Vietnam	F/34	8	Pos	Neg		
10	97.2	Vietnam	M/18	8	Pos	Pos		
11	99.3	Vietnam	M/89	10		Pos		

12	101.1	Vietnam	F/-	9		Pos		Pos
13	101.2	Vietnam	F/-	12		Pos		Pos
14	101.3	Vietnam	F/-	15	Pos	Pos		Pos
15	109.2	Vietnam	M/53	9	Pos	Pos		
16	110.2	Vietnam	F/23	9	Pos	Neg		
17	111.2	Vietnam	F/19	9	Pos	Pos	Pos	Pos
18	120.2	Vietnam	M/-	8	Pos	Pos		
19	120.3	Vietnam	M/-	11	Pos	Pos	Pos	Pos
20	121.3	Vietnam	M/46	8	Pos	Pos	Pos	Pos
21	122.3	Vietnam	F/22	10	Pos	Pos	Pos	Pos
22	124.3	Vietnam	F/-	10	Pos	Pos	Pos	Pos
23	128.3	Vietnam	F/31	10	Pos	Pos	Pos	Pos
24	129.2	Vietnam	F/16	8	Pos	Pos	Pos	Neg
25	130.2	Vietnam	F/11	8	Pos	Pos	Pos	Pos
26	131.2	Vietnam	M/20	9		Pos	Pos	Pos
27	135.2	Vietnam	F/45	9	Pos	Pos	Pos	Pos
28	140.3	Vietnam	F/44	10		Pos		Pos
29	147.1	Vietnam	M/19	12		Neg		
30	147.2	Vietnam	M/19	15		Neg		
31	147.3	Vietnam	M/19	18		Pos		
32	148.2	Vietnam	F/13	8		Pos		
33	155.2	Vietnam	M/37	8		Pos		
34	155.3	Vietnam	M/37	11	Pos	Pos	Pos	
35	157.2	Vietnam	F/37	8		Pos		
36	161.2	Vietnam	M/21	8		Pos	Pos	Pos
37	161.3	Vietnam	M/21	11	Pos	Pos	Pos	
38	172.2	Vietnam	M/32	10	Pos	Pos	Pos	
39	174.2	Vietnam	M/37	10	Pos	Pos	Pos	
40	185.3	Vietnam	M/54	8	Pos	Neg	Pos	
41	186.2	Vietnam	M/-	8	Pos	Pos	Pos	
42	187.3	Vietnam	F/34	9	Pos	Pos	Pos	
43	191.2	Vietnam	M/20	8		Pos		
44	208.2	Vietnam	M/15	8		Pos		
45	213.2	Vietnam	F/14	8		Pos		Pos
46	218.2	Vietnam	M/17	9		Neg		Neg
47	219.2	Vietnam	M/41	8		Pos		
48	224.2	Vietnam	M/20	8		Neg		
49	224.3	Vietnam	M/20	11		Neg		Neg
50	225.3	Vietnam	F/22	9		Neg		
51	229.3	Vietnam	F/18	9		Pos		
52	350/17†	Philippines	M/28		Pos	Pos	Pos	Pos
53	420/17†		F/23		Pos	Pos	Pos	Pos
54	3976/17†	India	F/26		Pos	Neg	Pos	Pos
55	3723/17†	Thailand	M/19		Pos	Neg	Pos	Pos
56	1333/17†	Thailand	F/55		Pos	Pos	Pos	Pos

57	1360/17†	Sri Lanka	F/41		Pos	Pos	Pos	Pos
58	1471/17†	Seychelles	F/55		Pos	Pos	Pos	Pos
59	1500/17†	Seychelles	M/65		Pos	Pos	Pos	Pos
60	1516/17†	Seychelles	M/81		Pos	Pos	Pos	

*Patients were initially confirmed positive for DENV-2 by qRT-PCR at the time of hospitalization. The samples analyzed in this study were obtained from follow-up blood draws collected more than 7 days after symptom onset.

†Israeli traveler

Table S3: Characteristics of JEV-positive samples (total number of samples $n = 28$). Abbreviations: F – female, M – Male, Pos – Positive, Neg – Negative

#	Patient #	Country of possible exposure	ELISA JEV-IgM	OMB anti-NS1 DENV-1 IgG
1	199/19	Vietnam	Pos	Pos
2	373/19	Vietnam	Pos	Neg
3	410/19	Vietnam	Pos	Neg
4	444/19	Vietnam	Pos	Neg
5	445/19	Vietnam	Pos	Neg
6	455/19	Vietnam	Pos	Neg
7	458/19	Vietnam	Pos	Neg
8	459/19	Vietnam	Pos	Neg
9	469/19	Vietnam	Pos	Neg
10	470/19	Vietnam	Pos	Neg
11	471/19	Vietnam	Pos	Neg
12	483/19	Vietnam	Pos	Neg
13	513/19	Vietnam	Pos	Neg
14	514/19	Vietnam	Pos	Neg
15	600/19	Vietnam	Pos	Pos
16	632/19	Vietnam	Pos	Neg
17	633/19	Vietnam	Pos	Neg
18	634/19	Vietnam	Pos	Neg
19	691/19	Vietnam	Pos	Neg
20	692/19	Vietnam	Pos	Neg
21	717/19	Vietnam	Pos	Neg
22	718/19	Vietnam	Pos	Pos
23	814/19	Vietnam	Pos	Pos
24	815/19	Vietnam	Pos	Neg
25	899/19	Vietnam	Pos	Neg
26	1097/19	Vietnam	Pos	Pos
27	1098/19	Vietnam	Pos	Pos
28	1099/19	Vietnam	Pos	Neg

Table S4: Characteristics of WNV-positive samples (total number of samples $n = 23$). Abbreviations: F – female, M – Male, Pos – Positive, Neg – Negative, ct – Cycle threshold.

#	Patient #	Gender / age(yr)	qRT-PCR result	ELISA WNV IgM	ELISA WNV IgG	OMB anti-NS1 DENV-1 IgG
1	1103/21	F/36	Pos	Pos	Pos	Neg
2	1528/20	M/57	Pos	Pos	Pos	Neg
3	1553/20	F/23	Pos	Pos	Pos	Pos
4	2014/20	F/35	Pos	Pos	Pos	Neg
5	1640/20	F/84	Pos	Pos	Pos	Neg
6	1710/20	F/21	Pos	Pos	Pos	Neg
7	1759/20	M/47	Pos	Pos	Pos	Neg
8	3350/19	F/18	Pos	Neg	Pos	Neg
9	3579/19	M/20	Pos	Pos	Pos	Neg
10	3455/18	F/51	Pos	Pos	Pos	Neg
11	3539/18	M/64	N.A	Pos	Pos	Pos
12	3708/18	M/66	Pos	Pos	Pos	Pos
13	5293/18	M/28	Pos	Pos	Pos	Neg
14	3126/18	F/79	Pos	Pos	Pos	Neg
15	3590/18	M/73	Pos	Pos	Pos	Pos
16	3631/18	F/45	Pos	Pos	Pos	Pos
17	3162/18	M/63	Pos	Pos	Pos	Pos
18	3291/18	M/86	N.A	Pos	Pos	Neg
19	3602/18	F/39	Pos	Pos	Pos	Neg
20	3794/18	F/47	N.A	Pos	Pos	Neg
21	1904/15	F/84	Neg	Pos	Pos	Pos
22	1750/15	M/35	Pos	Pos	Pos	Pos
23	1093/15	M/77	N.A	Pos	Pos	Neg

Table S5: Characteristics of ZIKV-positive samples[*] (total number of samples $n = 26$). Abbreviations: F – female, M – Male, Pos – Positive, Neg – Negative, Equ – Equivocal.

#	Patient #	Country of possible exposure	Gender / age(yr)	qRT-PCR result	Neutralization result (Titer)	ELISA Dengue		Time from onset (days)	ELISA Zika		OMB anti-NS1 DENV-1 IgG	OMB anti-NS1 DENV-3 IgG
						IgM	IgG		IgM	IgG		
1	514/16	Colombia	F/32	Pos	Pos (1280)	Neg	Pos	53	Equ	Pos	Pos	Neg
2	399/16	Dominican Republic	M/30	Pos	Pos (1280)	Neg	Pos	26	Pos	Pos	Pos	Pos
3	1643/16	Jamaica	M/23	Pos	Pos (640)	Neg	Neg	25	Pos	Pos	Neg	Neg

4	3185/16	Mexico	M/26	Pos	Pos (640)	Neg	Pos	27	Equ	Pos	Pos	Pos
5	3426/16	Mexico	M/37	Pos	Pos (1280)	Neg	Pos	5	Equ	Neg	Neg	Neg
6	40/17	Mexico	M/37	Pos	Pos (1280)	Neg	Pos	59	Neg	Pos	Pos	Neg
7	3988/16	Costa Rica	M/20	Neg	Pos (1280)	Neg	Pos	29	Pos	Pos	Pos	Pos
8	4343/16	Mexico/ Cuba	F/21	Pos	Pos (1280)	Neg	Neg	16	Pos	Pos	Pos	Pos
9	2516/16	Honduras	F/22	Neg	Pos (160)	Neg	Pos	~60	Neg	Pos	Pos	Pos
10	4361/16	Central America	M/29	Neg	Pos (320)	Neg	Neg	105	Neg	Pos	Pos	Pos
11	319/17	Panama	M/30	Neg	Pos (40)	Neg	Pos	~105	Neg	Pos	Neg	Neg
12	437/17	Panama	M/30	Neg	Pos (40)	Neg	Pos	~113	Neg	Equ	Neg	Pos
13	441/17	Cuba/ Mexico	M/29	Neg	Pos (320)	Neg	Neg	~75	Neg	Pos	Pos	Pos
14	1224/17	Thailand/ Philippines	M/26	Neg	Pos (160)	Neg	Pos	~30	Neg	Pos	Pos	Neg
15	2206/17	Porto-Rico	M/35	Neg	Pos (20)	Neg	Pos	~70	Neg	Pos	Neg	Pos
16	1021/19		F/53	Pos	Pos (160)				Neg	Pos	Pos	Pos
17	2190/19	Thailand	F/49	Pos	Pos (320)				Equ	Pos	Pos	Pos
18	3715/19		F/43	Pos	Pos (80)				Neg	Pos	Pos	Pos
19	3554/19		F/35	Pos	Pos (20)				Pos	Pos	Pos	Pos
20	Kamada 2	Thailand	M/27	Pos	Pos (40)	Pos	Neg		Pos	Neg	Neg	Neg
21	Kamada 3	Costa Rica/ Guatemala	M/27	Pos	Pos (1280)				Pos	Neg	Neg	Neg
22	Kamada 4	Dominican Republic	M/30	Pos	Pos (1280)				Pos	Pos	Pos	Pos
23	Kamada 5	Jamaica	M/23	Pos	Pos (640)				Pos	Equ	Neg	Neg
24	Kamada 6	Mexico/ Cuba	M/29	Neg	Pos (320)	Neg	Neg		Neg	Pos	Pos	Pos
25	Kamada 7	Colombia	F/50	Pos	Pos (640)				Pos	Pos	Neg	Neg
26	Kamada 8	Colombia	F/22	Neg	Pos (40)				Neg	Pos	Pos	Pos

*Patients were initially confirmed positive for ZIKV by either qRT-PCR or Neutralization tests at the time of hospitalization. The samples analyzed in this study were obtained from follow-up blood draws collected more than 5 days after symptom onset.

Table S6: Characteristics of SARS-Cov-2-positive samples* (total number of samples $n = 22$).

#	Patient #	Time from PCR test (days)	ELISA SARS-Cov-2 IgG	OMB anti-NS1 DENV-1 IgG
1	CO-7326	76	Pos	Neg
2	CO-7327	264	Pos	Neg
3	CO-7328	267	Pos	Neg
4	CO-7329	267	Pos	Neg
5	CO-7330	267	Pos	Neg
6	CO-7386	N.A.	Pos	Neg
7	CO-7387	N.A.	Pos	Neg
8	CO-7388	N.A.	Pos	Neg
9	CO-7389	N.A.	Pos	Neg
10	CO-7395	N.A.	Pos	Neg
11	CO-7396	N.A.	Pos	Neg
12	CO-7397	N.A.	Pos	Neg
13	CO-7401	N.A.	Pos	Neg
14	CO-7402	N.A.	Pos	Neg
15	CO-7404	72	Pos	Neg
16	CO-7407	101	Pos	Neg
17	CO-7408	275	Pos	Neg
18	CO-7410	259	Pos	Neg
19	CO-7411	150	Pos	Neg
20	CO-7412	79	Pos	Neg
21	CO-7416	N.A.	Pos	Neg
22	CO-7419	N.A.	Pos	Neg

*Patients were initially confirmed positive for SARS-CoV-2 by qRT-PCR at the time of hospitalization. The samples analyzed in this study were obtained from follow-up blood draws collected more than 7 days after symptom onset.

List of NS1 sequences used for BLAST analysis

(https://www.uniprot.org/uniprotkb/W8FM70/entry)

>DENV1-NS1_W8FM70_9FLAV

DSGCVINWKGRELKCGSGIFVTNEVHTWTEQYKFQADSPKRLSAAIGKAWEEGVCGIRSA
TRLENIMWKQISNELNHILLENDMKFTVVVGEVNGILAQGKKMIRPQPMEHKYSWKSWG
KAKVIGADVQNTTFIIDGPNTPECPDDQRAWNIWEVEDYGFGIFTTNIWLKLRDSYTQVCD
HRLMSAAIKDSKAVHADMGYWIESEKNETWKLARASFIEIKTCIWPKSHTLWSNGVLESE
MIIPKIYGGPISQHNYRPGYFTQTAGPWHLGKLELDFELCEGTTVVVDEHCGNRGPSLRTTT
VTGKIIHEWCCRSCTLPPLRFKGEDGCWYGMEIRPVKEKEENLVKSMVSA

(https://www.uniprot.org/uniprotkb/Q04040/entry)

>DENV2-NS1_Q04040_9FLAV

DSGCVVSWKNKELKCGSGIFITDNVHTWTEQYKFQPESPSKLASAIQKAHEEGICGIRSVTR
LENLMWKQITPELNHILSENEVKLTIMTGDIKGIMQAGKRSLRPQPTELKYSWKTWGKAK
MLSTESHNQTFLIDGPETAECPNTNRAWNSLEVEDYGFGVFTTNIWLKLKEKQDVFCDSKL
MSAAIKDNRAVHADMGYWIESALNDTWKIEKASFIEVKNCHWPKSHTLWSNGVLESEMII
PKNLAGPVSQHNYRPGYHTQITGPWHLGKLEMDFDFCDGTTVVVTEDCGNRGPSLRTTTA
SGKLITEWCCRSCTLPPLRYRGEDGCWYGMEIRPLKEKEENLVNSLVTA

(https://www.uniprot.org/uniprotkb/A0A2H4ZCQ2/entry)

>DENV3-NS1_A0A2H4ZCQ2_9FLAV

DMGCVINWKGKELKCGSGIFVTNEVHTWTEQYKFQADSPKRLATAIAGAWENGVCGIRST
TRMENLLWKQIANELNYILWENNIKLTVVVGDIIGVLEQGKRALTPQPMELKYSWKTWGK
AKIVTAEIQNSSFIIDGPNTPECPSASRAWNVWEVEDYGFGVFTTNIWLKLREVYTQTCDHR
LMSAAIKDERAVHADMGYWIESQKNGSWKLEKASFIEVKTCTWPKSHTLWSNGVLESDM
IIPKSLAGPISQHNHRPGYHTQTAGPWHLGKLELDFNYCEGTTVVITENCGTRGPSLRTTTV
SGKLIHEWCCRSCTLPPLRYMGEDGCWYGMEIRPINEKEENMVKSLVSA

(https://www.uniprot.org/uniprotkb/A0A2H4ZCQ3/entry)

>DENV4-NS1_A0A2H4ZCQ3_9FLAV

DMGCVVSWNGKELKCGSGIFVVDNVHTWTEQYKFQPESPARLASAILNAHKDGVCGIRST
TRLENVMWKQITNELNYVLWEGGHDLTVVAGDVKGVLTKGKRALTPPVNDLKYSWKT
WGKAKIFTPEARNSTFLIDGPDTSECPNERRAWNFFEVEDYGFGMFTTNIWMKFREGSSEV
CDHRLMSAAIKDQKAVHADMGYWIESSKNQTWQIEKASLIEVKTCLWPKTHTLWSNGVL
ESQMLIPRSYAGPFSQHNYRQGYATQTAGPWHLGKLEIDFGECPGTTVTIQEDCDHRGPSL
RTTTASGKLVTQWCCRSCTMPPLRFLGEDGCWYGMEIRPLSEKEENMVKSQVTA

(https://www.sinobiological.com/research/virus/zika-virus-ns1-amino-acid-sequence)

>ZIKV_A0A0U3FSM8_ZIKV

VGCSVDFSKKETRCGTGVFVYNDVEAWRDRYKYHPDSPRRLAAAVKQAWEDGICGISSV
SRMENIMWRSVEGELNAILEENGVQLTVVVGSVKNPMWRGPQRLPVPVNELPHGWKAW
GKSHFVRAAKTNNSFVVDGDTLKECPLKHRAWNSFLVEDHGFGVFHTSVWLKVREDYSL
ECDPAVIGTAVKGKEAVHSDLGYWIESEKNDTWRLKRAHLIEMKTCEWPKSHTLWTDGIE
ESDLIIPKSLAGPLSHHNTREGYRTQMKGPWHSEELEIRFEECPGTKVHVEETCGTRGPSLR
STTASGRVIEEWCCRECTMPPLSFRAKDGCWYGMEIRPRKEPESNLVRSMVTAGSTDHMD
HFSL

(https://www.uniprot.org/uniprotkb/A0A2L1IPF6/entry#sequences)

>West_Nile_virus_A0A2L1IPF6

DTGCAIDISRQELRCGSGVFIHNDVEAWMDRYKYYPETPQGLAKIIQKAHKEGVCGLRSVS
RLEHQMWEAVKDELNTLLKENGVDLSIVVEKQEGMYKSAPRRLTATTEKLEIGWKAWGK
SILFAPELANNTFVIDGPETKECPTQNRAWNSLEVEDFGFGLTSTRMFLRVRESNTTECDSKI

IGTAVKNNLAIHSDLSYWIESRFNDTWKLERAVLGEVKSCTWPETHTLWGDGVLESDLIIPI
TLAGPRSNHNRRPGYKTQSQGPWDEGRVEIDFDYCPGTTVTLSESCGHRGPATRTTTESGK
LITDWCCRSCTLPPLRYQTDNGCWYGMEIRPQRHDEKTLVQSQVNA

(https://www.ncbi.nlm.nih.gov/protein/NP_775667.1?report=fasta)
>NP_775667.1 non-structural protein NS1 [Japanese encephalitis virus]
DTGCAIDITRKEMRCGSGIFVHNDVEAWVDRYKYLPETPRSLAKIVHKAHKEGVCGVRSV
TRLEHQMWEAVRDELNVLLKENAVDLSVVVNKPVGRYRSAPKRLSMTQEKFEMGWKA
WGKSILFAPELANSTFVVDGPETKECPDEHRAWNSMQIEDFGFGITSTRVWLKIREESTDEC
DGAIIGTAVKGHVAVHSDLSYWIESRYNDTWKLERAVFGEVKSCTWPETHTLWGDGVEE
SELIIPHTIAGPKSKHNRREGYKTQNQGPWDENGIVLDFDYCPGTKVTITEDCGKRGPSVRT
TTDSGKLITDWCCRSCSLPPLRFRTENGCWYGMEIRPVRHDETTLVRSQVDA
